# Glycosylphosphatidylinositol anchor biosynthesis pathway-based biomarker identification with machine learning for prognosis and T cell exhaustion status prediction in breast cancer

**DOI:** 10.3389/fimmu.2024.1392940

**Published:** 2024-07-02

**Authors:** Haodong Wu, Zhixuan Wu, Hongfeng Li, Ziqiong Wang, Yao Chen, Jingxia Bao, Buran Chen, Shuning Xu, Erjie Xia, Daijiao Ye, Xuanxuan Dai

**Affiliations:** ^1^ Department of Breast Surgery, Key Laboratory of Clinical Laboratory Diagnosis and Translational Research of Zhejiang Province, the First Affiliated Hospital of Wenzhou Medical University, Wenzhou, Zhejiang, China; ^2^ School of Molecular Science, University of Western Australia, Perth, WA, Australia; ^3^ Department of Computer Information Systems, Georgia State University, Atlanta, GA, United States

**Keywords:** breast cancer disease, glycosylphosphatidylinositol anchor biosynthesis, T cell exhaustion, pan-cancer, scRNA-seq, machine learning, diagnosis

## Abstract

As the primary component of anti-tumor immunity, T cells are prone to exhaustion and dysfunction in the tumor microenvironment (TME). A thorough understanding of T cell exhaustion (TEX) in the TME is crucial for effectively addressing TEX in clinical settings and promoting the efficacy of immune checkpoint blockade therapies. In eukaryotes, numerous cell surface proteins are tethered to the plasma membrane via Glycosylphosphatidylinositol (GPI) anchors, which play a crucial role in facilitating the proper translocation of membrane proteins. However, the available evidence is insufficient to support any additional functional involvement of GPI anchors. Here, we investigate the signature of GPI-anchor biosynthesis in the TME of breast cancer (BC)patients, particularly its correlation with TEX. GPI-anchor biosynthesis should be considered as a prognostic risk factor for BC. Patients with high GPI-anchor biosynthesis showed more severe TEX. And the levels of GPI-anchor biosynthesis in exhausted CD8 T cells was higher than normal CD8 T cells, which was not observed between malignant epithelial cells and normal mammary epithelial cells. In addition, we also found that GPI -anchor biosynthesis related genes can be used to diagnose TEX status and predict prognosis in BC patients, both the TEX diagnostic model and the prognostic model showed good AUC values. Finally, we confirmed our findings in cells and clinical samples. Knockdown of PIGU gene expression significantly reduced the proliferation rate of MDA-MB-231 and MCF-7 cell lines. Immunofluorescence results from clinical samples showed reduced aggregation of CD8 T cells in tissues with high expression of GPAA1 and PIGU.

## 1 Introduction

Nowadays, breast cancer, as the most prevalent malignant tumor in women, has replaced lung cancer as the cancer with the highest incidence rate worldwide (1). Although numerous advances in early screening, and diagnostic methods, the incidence of BC is increasing yearly, constitutes ~11.7% of all new cancer patients (2). Therefore, comprehending the involvement of diverse oncogenes and risk factors in the initiation and progression of BC assumes significance for early prevention and treatment strategies targeting this disease.

Due to the distinctive immunostimulatory properties exhibited by breast cancer, immunotherapy has gained considerable attention as a promising therapeutic approach for breast cancer (3). In the context of immunotherapeutic therapy, immune cells play a pivotal role as the cellular basis. In certain highly immune-infiltrated tumors, tumor-infiltrating lymphocytes (TILs) can constitute more than 40% of the overall cell population (4). The functional status of T cells is crucial for tumor evasion and immune therapy. However, the benefits of immunotherapy in cancer treatment still remains limited due to T cell functional dysregulation. It is typically characterized by reduced proliferation capacity and impaired effector function of T cells, as well as the excessive expression of various inhibitory receptors (5), which result in a weakened immune response and the escape of tumor cells from immune surveillance (6). CD8 T cell exhaustion, which epitomizes the dysfunctional state of T cells within the TME, is widely recognized as a major obstacle to current anticancer immunotherapy (7).It is imperative to comprehensively investigate the development mechanisms underlying T cell exhaustion in TME and devise novel strategies to avert functional T cell exhaustion in immunotherapeutic approaches.

Glycosylphosphatidylinositol (GPI) anchor biosynthesis is one of the generally exists in eukaryotic protein modification after translation process (8). In simple terms, it provides a “glycosylphosphatidylinositol anchor” for certain intracellular proteins to specifically anchor onto the lipid bilayer (9), thereby enabling their precise localization and engagement in vital biological processes such as cellular signaling, adhesion, and immune responses (10).. Abnormalities in the processes of GPI-anchor biosynthesis have been shown to be involved in a variety of diseases, such as paroxysmal nocturnal hemoglobinuria and the syndrome of hyperphosphatemia with mental retardation (11, 12). In addition, more and more studies have found that there are identified abnormalities of GPI-anchor biosynthesis in tumorigenesis and development, including the dysregulated expression of genes involved in GPI biosynthesis and increased synthesis of specific GPI-anchored proteins (GPI-AP) (13). The biosynthesis of GPI-anchored proteins has been found to be closely associated with the regulation of T cell functions in the immune response (14). Studies indicate that GPI-anchored proteins play crucial roles in regulating T cell activation, proliferation, and cell death processes (15). In conditions such as chronic infections, tumor microenvironments, or autoimmune diseases, T cells may undergo functional exhaustion, leading to immune response suppression and reduced therapeutic efficacy (16). These findings suggest that the components of GPI-anchor biosynthesis and the high expression of tumor-associated GPI-AP may play a role in carcinogenesis, providing new ideas for the exploration of tumor markers and targeted therapies.

In the context of breast cancer, the biosynthesis of GPI-anchored proteins has also been found to be closely associated with the proliferation, invasion, and metastasis of breast cancer cells (17, 18). By intervening in the GPI-anchored biosynthesis pathway, we may potentially impact the survival and invasiveness of breast cancer cells, thereby enhancing treatment efficacy and patient survival rates. In this study, we aimed to explore the link of GPI anchor biosynthesis with T cell exhaustion and tumor prognostic progression in BC.

## 2 Materials and methods

### 2.1 Collection of raw data

The RNA-seq data and clinical information of patients were obtained from the TCGA and GTEx databases (http://xena.ucsc.edu/). The BC cohort contains 1097 cancer samples and 120 paracancer samples. The pan-cancer dataset contains 10,534 samples covering 33 different types of cancers. All the TCGA files are stored in FPKM standard format. The validation dataset was downloaded from the Metabric database (https://www.cBioPortal.org), which provides RNA-seq data and related clinical information of 1918 BC patients. In addition, we downloaded the scRNA-seq profiles of BC from the GEO database (https://www.ncbi.nlm.nih.gov/GSE114727) and selected 11 normal breast single-cell samples and 30 tumor single-cell samples from it for subsequent analysis.

### 2.2 Obtaining the necessary genes for various scoring systems

The Glycosylphosphatidylinositol (GPI) - anchor biosynthesis related genes were obtained from the relevant literature as indicated in **Supplementary Table 1** (19). The scoring genes for cytotoxic T lymphocytes (CTL) are sourced from the String database (https://cn.string-db.org/) (**Supplementary Table 2**). The chemokine score consists of 41 known chemokines (**Supplementary Table 3**). All the scores are determined by the expression of scoring genes with the ssGSEA Algorithm (20). The cytolytic activity score (CYT-score), which quantifies the cell-killing function, is based on the geometric mean of gene expressions of GZMA and PRF1 (21).

### 2.3 Pan-cancer prognostic analysis of GPI- anchor biosynthesis score

Package ‘GSVA’, was employed to calculate GPI-anchor biosynthesis score (GPI-score) for patients in the TCGA pan-cancer dataset. Differences in GPI-scores between various tumors and normal samples were assessed using the “ggpubr” software package. The association between GPI-scores and prognostic indicators of patients was investigated by univariate Cox regression analysis (uniCox).Prognostic indicators include: overall survival (OS); disease-free interval (DFI); progression-free interval (PFI); and disease-specific survival (DSS) (22). And the Kaplan-Meier method was employed to assess differences in OS across distinct groups (23).

### 2.4 Functional enrichment analysis

The differential gene analysis among various groups was conducted using the “limma” package, and | log2 fold change (FC) |≥ 0.5 and p< 0.05 were the standard for the differential genes. Finally, KEGG and GSEA enrichment analysis of the differential genes was performed on SangerBox portal (https://www.sangerbox.com) (24).

### 2.5 Immune microenvironment analysis

Apply the “ssGSEA” algorithm to assess the enrichment scores of 22 immune cell types in every individual, while employing the deconvolution method with LM22 immunological feature matrix in CIBERSORT for precise calculation of infiltrating immune cell components (25). In addition, Stromal score and immune score were calculated by “ESTIMATE” algorithm (26).

### 2.6 Consensus clustering

Previous studies have demonstrated that interleukin-2 (IL-2), tumor necrosis factor (TNF), interferon-gamma (IFN-γ), and the cytotoxic T lymphocyte (CTL) can serve as quantitative markers for assessing the abundance of T cell exhaustion (27, 28). Consensus clustering analysis was performed on the TCGA BC cohort based on the aforementioned characteristics of T cell exhaustion with K-Means clustering algorithm. The R package “ConsensusClusterPlus” was used to determine the number of clusters with the parameters were set as reps=50, maxK=9, pFeature=1, pItem=0.8. And 1000 repetitions were carried out to ensure the stability of the subtype (29).

### 2.7 Processing of the scRNA-seq data

The “Seurat” package was used for quality control of data, which involved excluding cells exhibiting ≥5% expression of mitochondria-related genes and those with fewer than 50 detected genes. Further normalization of the gene expression matrix is conducted based on the top 3000 variable genes. The significant principal components (PCs) were computed by principal component analysis (PCA), and the dimensionality was subsequently reduced for the first 20 PCs. Then, a cluster classification analysis was conducted on all units. With the help of the CellMarker database and FindAllMarkers function, we annotated different cell clusters. There are significant differences in the chromosomal copy number variations (CNV)between malignant cells and normal cells in solid tumors. The InferCNV algorithm is widely used for exploring scRNA-seq data to provide evidence of large-scale chromosomal copy number alterations, thereby effectively identifying malignant cells (30). We utilized the InferCNV algorithm to identify malignant cells within clusters of epithelial cells. The GPI-score in different cell clusters was calculated by the AddModuleScore algorithm implemented in Seurat. The Monocle 2 algorithm was employed for pseudo-time trace analysis, and the plot_pseudotime_heatmap function was utilized to construct heatmaps illustrating the dynamic expression of genes (31).

### 2.8 Establishment of a diagnostic model for T cell exhaustion

We performed ssGSEA analysis on the characteristics of T-cell exhaustion (including IL2, TNF, IFNG, TGFB1, IL10, TCF7, TBX21, TOX expression abundance, CTL score, Chemokines score, and CYT score) to derived a T-cell exhaustion score (TEX-score). Based on the median TE-score, we divided BC patients into two types (Severe exhaustion type and mild exhaustion type). Different exhaustion states are included as the outcomes of the model. The XGBoost algorithm and the Logistic regression algorithm were used for model training, and the TEX-score of patients was used as the gold standard to evaluate the predictive value of these two models for T-cell exhaustion. The ROC analysis for outcome prediction was conducted using pROC’s ROC function, and the final AUC result was determined by utilizing pROC’s ci function to obtain both AUC and confidence intervals.

### 2.9 Construction of a prognostic model for breast cancer

Integrating GPI-anchor biosynthesis related genes and T cell exhaustion related genes (IL2, TNF, IFNG) as input features, we arranged 101 combinations of 10 algorithms in the training dataset for variable selection and model construction based on a ten-fold cross-validation framework (32). Optimal modeling method selection is based on c-din values, followed by feature gene screening, calculation of the proportion of feature genes in the prognostic model, and subsequent construction of a risk score.


$$\text{Risk score = }\sum_{n}\text{Coefficient of gene (n)}\times \text{Expression of gene (n)}$$

### 2.10 Immunofluorescence and cellular experiment

25 BC patients were recruited from the First Affiliated Hospital of Wenzhou Medical University (**Supplementary Table 4**). Breast tumor tissue was processed into 4μm thick paraffin sections and subsequently dewaxed. The sections were then incubated in 0.01M sodium citrate at room temperature for 100 minutes to eliminate endogenous enzyme activity. Subsequently, a solution containing 5% goat serum was applied and incubated at room temperature for 60 minutes, followed by overnight incubation with DAPI (1:100), CD8 (1:100), PIGU (1:100), and GPAA1 (1:100) antibodies, respectively. On the following day, after washing with 0.02% PBST, a secondary antibody solution (concentration of 1:200) was added under light-avoiding conditions and incubated at room temperature for 2 hours. Finally, the sections were washed with 0.02% PBST, mounted, and observed using confocal optical microscopy to visualize the staining results.

Small interfering RNA (siRNA) was designed by Tongyong (Shanghai)Company to knock down PIGU mRNA in BRCA cell lines. Total RNA was extracted by Trizol reagent (RNAiso Plus-9109, Takara), and cDNA was synthesized through PCR thermal cycler (Eppendorf, Germany) using reverse transcription reagent (RR036A, Takara) according to the instructions. An appropriate amount of cDNA was used as a template to verify the efficiency of knock down for qRT-PCR through TB Green dye (RR820A, Takara) on Mastercycler real-time PCR system (LightCycler, Roche). After targeted gene knock down, siNC and siPIGU cells were seeded in 96-well plates and cultivated for 48h. CCK8 reagent (CK04, Dojindo) was added and incubated for 2h. The absorbance was measured at 450nm by microplate reader (Tecan, Switzerland).

### 2.11 Statistical analysis

The t-test was applied to the normally distributed data in this study, while the Wilcoxon rank sum test was used for analyzing the non-normally distributed data. The chi-square test was employed to compare categorical variables. In order to compare the disparities among small sample sizes, a non-parametric test is selected. Pearson correlation analysis is used to measure the strength and direction of the linear relationship between two variables. All the analysis were conducted on the R software (version 4.1.2).And adhering to standard practice, the results are statistically significant with p< 0.05.

## 3 Result

### 3.1 Glycosylphosphatidylinositol (GPI) anchor biosynthesis is a prognostic risk factor in breast cancer

**Figure 1:**
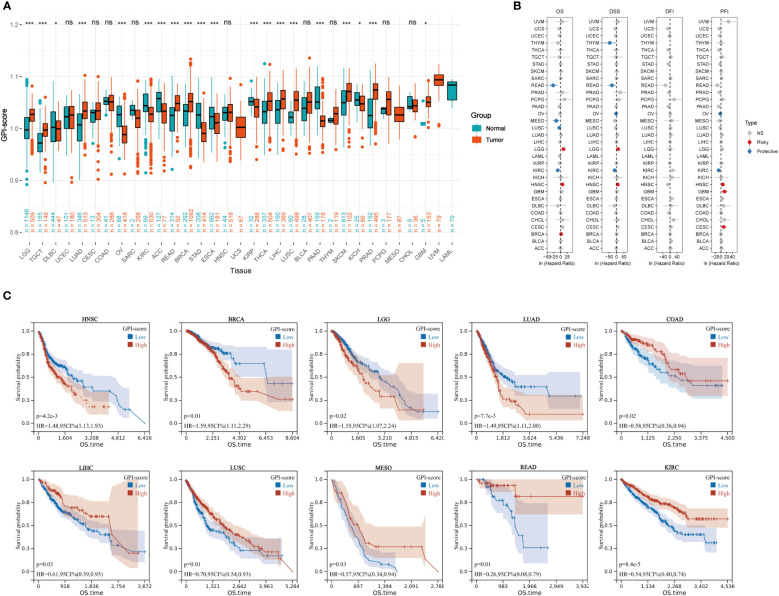
Prognostic analysis of GPI-score in multiple cancer types. **(A)** The boxplot shows GPI-score in 33 tumor types from TCGA and GTEx databases (ns, p ≥ 0.05; *p< 0.05; **p< 0.01; ***p< 0.001). **(B)** Univariate Cox regression analysis of GPI-scores with OS, DSS, PFI and DFI in patients with different cancers. **(C)** Kaplan–Meier (KM) curves of 10 cancer types (COAD, BRCA, LGG,LUAD, LIHC,HNSC,LUSC,MESO,READ and KIRC) in TCGA with significant survival differences.

We quantified the intensity of GPI-anchored biosynthesis in patients with GPI-scores and evaluated the differences in GPI-anchored biosynthesis between cancer and paracancer tissues in the TCGA pan-cancer dataset. **Figure 1A** shows that there are 20 cancer types exhibit significant changes in GPI-anchored biosynthesis. The levels of GPI-anchored biosynthesis in the tumor tissues of LGG, TGCT, LUAD, READ, BRCA, THCA, LIHC, LUSC, SKCM, PRAD and GBM were higher than those in normal tissues. Conversely, the levels of GPI-anchored biosynthesis in the tumor tissues of DLBC, OV, KIRC, KICH, STAD, ESCA, KIRP, PAAD and ACC were lower than normal tissues. As the result of unicox analysis shown in the forest diagram (**Figure 1B**), GPI-anchored biosynthesis is a risky factor of prognosis in LGG, HNSC, GBM, CESC and BRCA, while it is a protective factor in READ, THYM, OV, MESO, LUSC and KIRC. The results from the KM plotter demonstrated that the enhancements to the GPI-anchored biosynthesis was associated with poor prognosis in HNSC, BRCA, LGG and LUAD (**Figure 1C**). However, in COAD, LIHC, LUSC, MESO, READ and KIRC, the stronger the GPI-anchored biosynthesis, the more favorable the prognosis for patients. Combined the results of the cox regression, K-M survival curve and the expression of GPI-scores in tumor and normal samples, we finally determined that the GPI-anchored biosynthesis may exert a pivotal role in the development of breast cancer.

### 3.2 Functional enrichment analysis of the GPI-anchored biosynthesis

**Figure 2:**
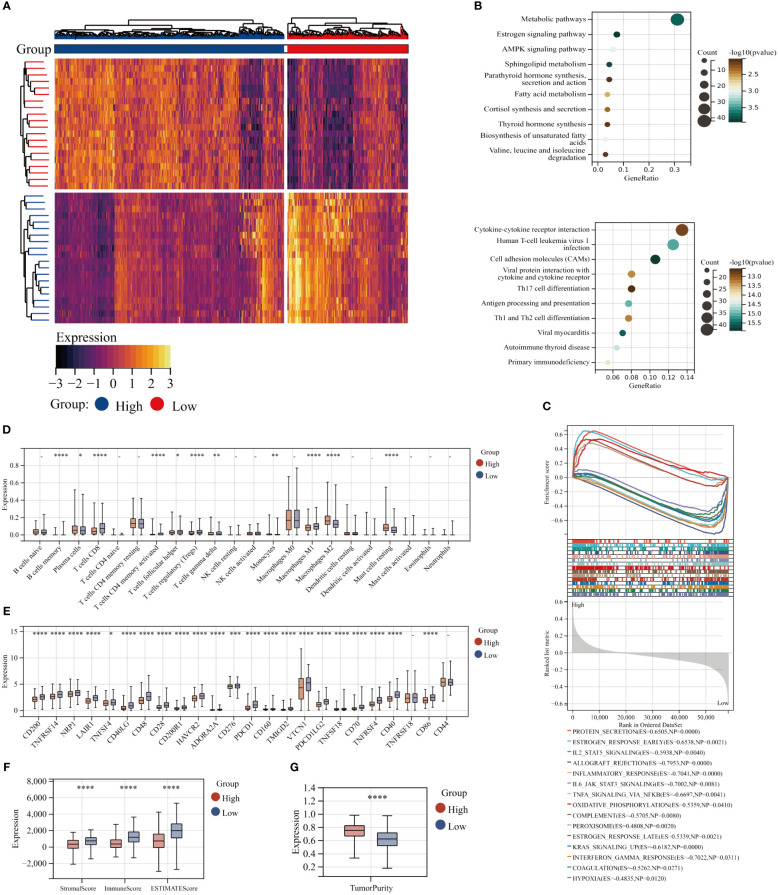
Functional enrichment analysis of the GPI-anchored biosynthesis. **(A)** Heat maps of Differential gene in patients with high and low GPI-score(|log2 fold change (FC)|≥1 and p< 0.05). **(B)** KEGG enrichment analyses of Up-regulated differential genes(up) and down-regulated differential genes(down). **(C)** GSEA analysis, the first 15 statistically significant pathways were presented. **(D)** The abundance of 22 immune cells in patients with high and low GPI-score from TCGA databases with CIBERSORT. **(E)** Expression of 24 immune checkpoints in different subgroups. **(F, G)** Immune infiltration scores and Tumor Purity score for different groups. (*p< 0.05; **p< 0.01; ***p< 0.001; ****p< 0.0001).

The TCGA BC patients were divided into two cohorts with high and low GPI-anchored biosynthesis based on the best cutoff values of the GPI-score. The heat map illustrates the distinct gene patterns within various patient subgroups (**Figure 2A**). KEGG functional enrichment analysis of different clusters of patients showed that the patients with high GPI-anchored biosynthesis had a worse prognosis accompanied by a lower immune response, the specific manifestations include the down-regulation of Cytokine-cytokine receptor interaction, Helper T lymphocytes (Th17, Th1 and Th2) cell differentiation, Primary immunodeficiency, and Antigen processing and presentation (**Figure 2B**).Interestingly, the patients in the high GPI-anchored biosynthesis group exhibited augmented metabolic pathways, characterized by the Estrogen signaling pathway and AMPK signaling pathway, as well as the enhanced Fatty acid metabolism and Cortisol synthesis secretion (**Figure 2B**). In addition, GSEA analysis showed that as the GPI-score increased, protein secretion and estrogen response were elevated, peroxisome metabolism and oxidative phosphorylation pathway were enhanced. However, IL2-STAT5 signaling, IL6-JAK-STAT3 signaling, TNFA signaling, Interferon Gamma response and Inflammatory response were downregulated (**Figure 2C**). We also examined the abundance of 22 immune cells in different groups. Surprisingly, the group of patients with high GPI-score showed a decrease in adaptive immune function, with a decrease in T cells CD8 ,T cells CD4 memory activated, T cells follicular helper, T cells regulatory (Tregs),and T cells gamma delta. It was also accompanied by an increasing of M2 macrophages (tumor-promoting) and monocytes (**Figure 2D**) (33). Simultaneously, the analysis of ESTIMATE scores and immune checkpoints revealed that patients with higher GPI-anchored biosynthesis exhibited a diminished immune status and response (**Figure 2E**). Additionally, these patients exhibited decreased stromal cell infiltration in their tumor tissues but elevated levels of tumor purity (**Figures 2F, G**).

### 3.3 The correlation between GPI-anchored biosynthesis and T-cell exhaustion

**Figure 3:**
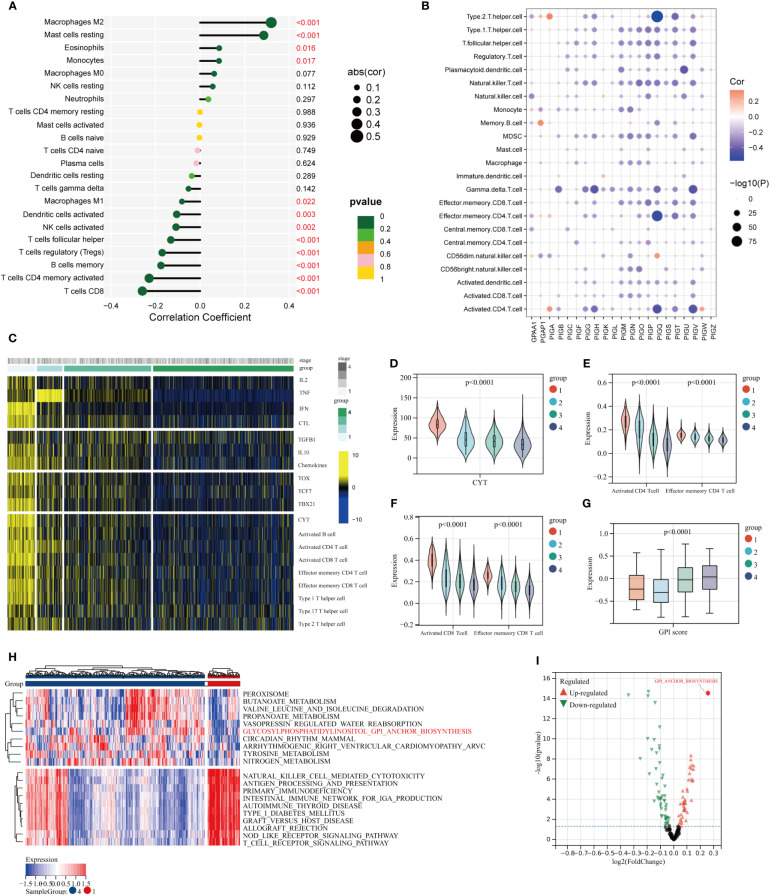
Characterization of TEX heterogeneity within the TME of BC. **(A)** The correlation between GPI-score and the abundance of 22 immune cells. **(B)** The correlation of GPI-anchored biosynthesis related genes with immune cells. **(C)** TCGA BC patients were divided into four subgroups according to the specific TEX pathway. **(D)** CYT scores for the four clusters. **(E, F)** Boxplots depict the relative abundance of infiltrating T lymphocytes across distinct clusters. **(G)** Expression of GPI-score among the four TEX subgroups. **(H)** Heat maps showing the first 10 differential pathways in patients with C1 and C4 clusters. **(I)** The volcano plot displays the log fold change of differential pathways in patients from C1 and C4 clusters.

GPI-score is negatively correlated with T cell CD8, T cell CD4 memory activation, Treg, B cell memory, and T-cell follicular helical (P<0.001), while showing a positive correlation with M2 macrophages (P<0.001) (**Figure 3A**). As it is depicted in **Figure 3B**, the expression of the majority of genes involved in GPI-anchored biosynthesis exhibited a significant negative correlation with diverse immune cell populations. Then, consensus clustering analysis was employed to categorize BC patients based on the specific TEX pathway which includes TNF, IL-2, IFN-γ, and CTL. We identified 4 clusters of patients with different T-cell exhaustion status in the TCGA-BC cohort (C1-C4) (**Supplementary Figure 1**). The heat map also shows the change of other molecular pathways of TEX in different groups, including TGFB, IL10, chemokines, transcription factors (TCF7, TBX21, Tox), CYT scores, and the abundance of infiltrating lymphocytes (Activated B cell, Activated CD4 T cell, Activated CD8 T cell, Effector memeory CD4 T cell, Effector memeory CD8 T cell, Th1, Th2, and Th17) (**Figure 3C**). Consistently, these characterized pathways of TEX including CYT and CD4 T cell and CD 8 T cell showed a decreasing trend in C1-C4 (**Figures 3D–F**), suggesting that the four TEX subgroups identified in this study accurately represent the biology of the TEX grading stage. Interestingly, the intensity of GPI-anchored biosynthesis increases with the severity of the TEX (**Figure 3G**). Based on the KEGG enrichment analysis, we compared the differences in biological function between the groups with the greatest differences in the degree of TEX (C1 and C4), and surprisingly, alterations in GPI-anchored biosynthesis were the most significant change between the two groups, topping the list in terms of magnitude of change (**Figures 3H, I**).

### 3.4 Single-cell analysis of GPI-anchored biosynthesis in TME

**Figure 4:**
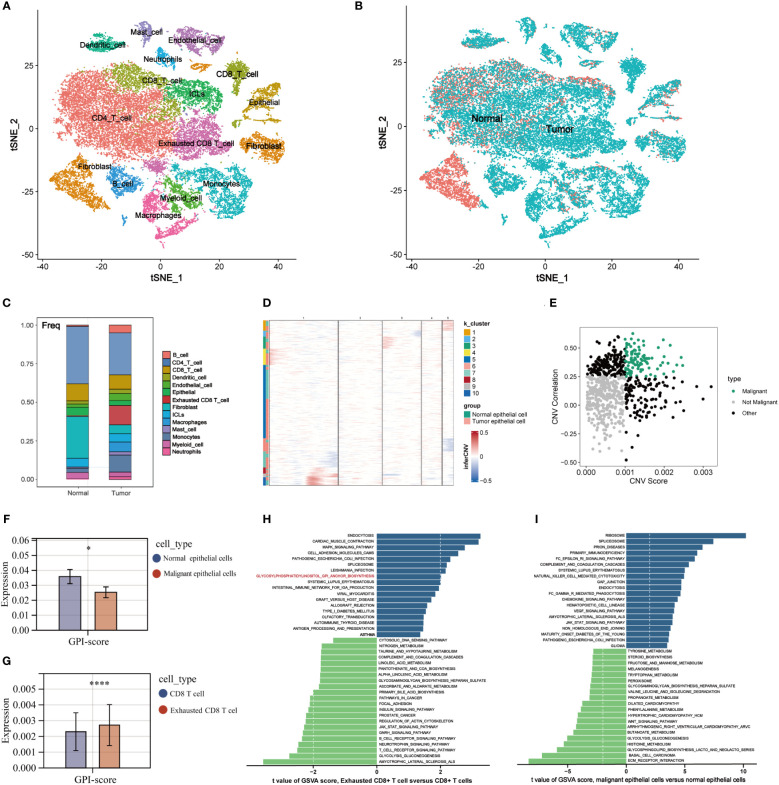
The signature of GPI-anchored biosynthesis with Single-cell analysis in TME of BC. **(A, B)** Dimensional reduction plot of single cells **(A)** 16 cell types, **(B)** tissue origins (tumor, normal). **(C)** The proportion of different types of cells in TME. **(D)** Identification of malignant epithelial cells by InferCNV algorithm. The CNV of epithelial cells in cluster 1, 3, 4, 8, 9, and 10 was significantly increased, and the tumor-derived epithelial cells in these clusters were defined as malignant epithelial cells. **(E)** Two-dimensional dot plot of malignant epithelial cells, non-malignant epithelial cells, and others. **(F, G)** Expression levels of GPI-score in different cell populations. (*p< 0.05; ****p< 0.0001) **(H, I)** Differential analysis of KEGG biological pathways in different cell populations.

We identified 14 major cell types using annotations from the Cell Marker database, including CD4 T cells, CD8 T cells, Exhausted CD8 T cells, B cells, Epithelial cells, Fibroblasts, Myeloid cells, Innate Lymphocytes (ILCs), Dendritic cells, Monocytes, Neutrophils, Mast cells, Endothelial cells and Macrophages (**Figure 4A**). Projecting the clinical information of the samples onto various cell subpopulations to trace the cellular origin, the exhausted CD8 T cells were predominantly present in tumor tissues (**Figure 4B**). Compared with the normal mammary microenvironment, the proportion of various types of cells in TME was significantly altered, in which the proportion of B cells, endothelial cells, macrophages, and monocytes was elevated while the CD4 T cells and CD8 T cells were decreased and replaced by a substantial increase in exhausted CD8 T cells (**Figure 4C**). This observation suggests that the T-cell exhaustion is a rare occurrence in normal breast tissue, but frequently manifests in tumor tissue. Then, we defined 127 malignant cells in the tumor epithelial cell population using the InferCNV algorithm (**Figures 4D, E**; **Supplementary Table 5**) and compared the differences of GPI-anchored biosynthesis between the malignant epithelial cells and normal breast epithelial cells. Interestingly, the GPI-anchored biosynthesis was instead reduced in malignant epithelial cells (**Figure 4F**). We compared the alterations of GPI-anchored biosynthesis in other tumor-derived cell populations, we found that remarkably differences could be only observed between CD8 T cells and exhausted CD8 T cells (**Figure 4G**; **Supplementary Figure 2**). The GPI-anchored biosynthesis was much stronger in exhausted CD8 T cells compared to normal CD8 T cells. Similarly, GSVA analysis of KEGG biological pathways showed there is no difference in GPI-anchored biosynthesis between malignant and non-malignant epithelial cells (**Figure 4H**), whereas this difference was evident in CD8 T cells and exhausted CD8 T cells (**Figure 4I**).

### 3.5 Pseudotime analysis of GPI-anchored biosynthesis related genes in TME

**Figure 5:**
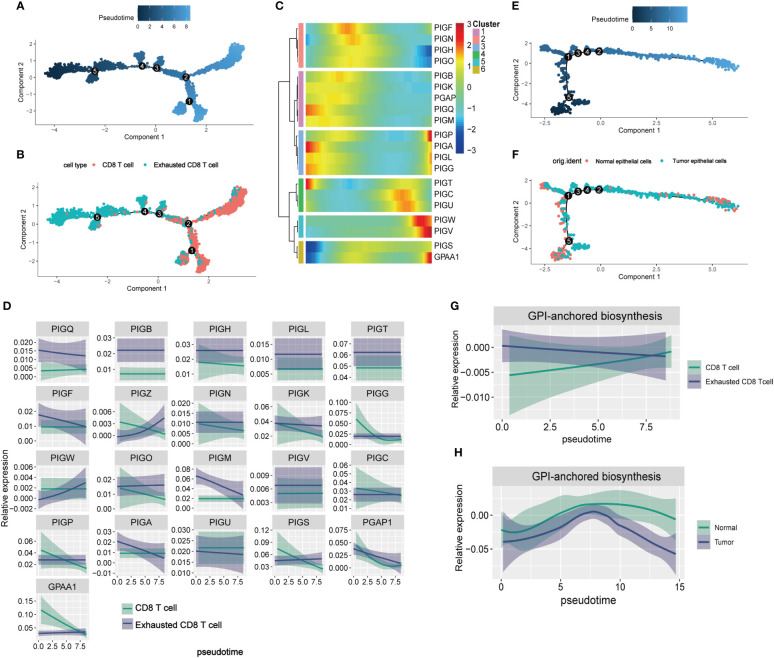
The pseudotime analysis of GPI-anchored biosynthesis related genes in TME. **(A, B)** Differentiation trajectory of CD8 T cells, colorcoded for pseudotime **(A)** and sub-cell types **(B)**. The direction of cell differentiation was CD8 T cells to exhibited CD8 T cells. **(C)** Pseudo-heatmap of genes altered in the differentiation process of CD8 T cells in BC **(D)** Dynamic changes of the expression of 21 GPI-anchored biosynthesis related genes in CD8 T cells and exhibited CD8 T cells. **(E, F)** Differentiation trajectory of epithelial cells in BC, colorcoded for pseudotime **(E)** and sub-cell types **(F). (G, H)** Two-dimensional plots showing the dynamic expression of GPI-anchored biosynthesis score during the T cell transitions **(G)** and epithelial cells transitions **(H)** along the pseudotime.

The differentiation trajectories of CD8 T cells and epithelial cells were constructed to observe the process of CD8 T cell exhaustion and metabolic remodeling of GPI-anchored biosynthesis during tumorigenesis. In the pseudotiming analysis, CD8 T cells were initially located along the trajectory path and gradually transitioned into exhausted CD8 T cells (**Figures 5A, B**). We calculated the dynamic expression levels of GPI-anchored biosynthesis related genes in CD8 T cells and exhausted CD8 T cells along the pseudotemporal transition, and the gene heat map suggested that PIGP, PIGC, PIGU, PIGW, PIGV, and GPAA1 were highly expressed at the end of the trajectory path (**Figure 5C**), which means that these genes are highly expressed in exhausted CD8 T cells. Two-dimensional map shows the dynamic expression of genes in CD8 T cells (green) and exhausted CD8 T cells (blue) (**Figure 5D**). These results suggest that the expression of GPI-anchored biosynthesis related genes is heterologous during the CD8 T cell state transition. **Figures 5E, F** represent the pseudotemporal developmental trajectories of epithelial cells. We calculated the GPI-scores in the cells and present them in a two-dimensional plot, where we can see that the overall level of GPI-anchored biosynthesis in exhausted CD8 T cells is always higher than that in non-exhausted CD8 T cell, and that metabolic intensity increases as CD8 T cell become more exhausted (**Figure 5G**). However, the intensity of GPI-anchored biosynthesis is lower in tumor epithelial cells than in normal epithelial cells (**Figure 5H**). Thus, it is worth considering whether GPI-anchored biosynthesis related genes could serve as a potential biomarker for predicting T cell exhaustion in BC patients. Interestingly, we did not observe this phenomenon in the metabolic landscape of exhausted CD4 T cells. It is worth noting that GPI-anchored biosynthesis levels are lower in exhausted CD4 T cells compared to non-exhausted CD4 T cells (**Supplementary Figure 3**).

### 3.6 Machine learning-based model for the diagnosis of T cell exhaustion

**Figure 6:**
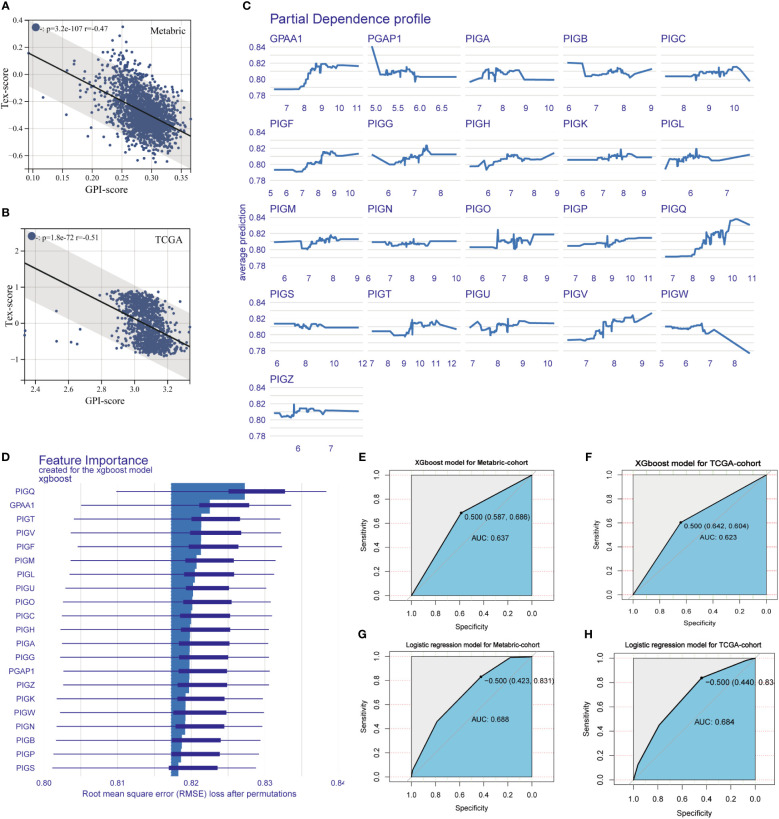
Machine learning-based models for the diagnosis of T cell exhaustion. **(A, B)** The correlation between GPI-score and Tex-score in Metabric (Spearman, r = -0.47, p = 3.2e-107) and TCGA BC queue (Spearman, r = - 0.51, p = 1.8e-72). **(C)** Partial Dependence Plot of XGboost model, part of the graph shows the dependence of the change in T-cell exhaustion prediction on each gene variable. **(D)** The importance of the features ranked in descending order. **(E-H)** The ROC curve were established following XGBoost and Logistic regression fed by the GPI-anchored biosynthesis related genes in Metabric and TCGA BC queue.

Correlation analysis was performed between the GPI-score and TEX-score in Metabric and TCGA BC cohort, with correlation coefficients (r-values) of -0.49 and -0.51 respectively (**Figures 6A, B**). Then, a variational analysis was performed in Metabric BC cohorts with the model_profile function in the XGBoost algorithm, assessing the association between the expression of GPI-anchored biosynthesis related genes and the states of T cell exhaustion in BC patients. The Partial Dependence profile demonstrates the impact of individual gene expression on the predictive ability of the XGboost model (**Figure 6C**). **Figure 6D** illustrates the magnitude of the impact of each GPI-anchored biosynthesis related gene on the states of T cell exhaustion in Metabric BC patients, it can be observed that PIGQ exerts the most significant influence on severe T cell exhaustion states in patients, followed by GPAA1. Finally, XGBoost and Logistic Regression were used to construct a T-cell exhaustion diagnostic model associated with GPI-anchored biosynthesis related genes in two BC cohorts. We evaluated the accuracy of these two models in predicting the occurrence of severe T-cell exhaustion in patients. The area under the ROC curve represe**n**ts the accuracy of the model. Compared to the XGBoost algorithm, the Logistic regression model has greater AUC values in both the Metabric and TCGA cohorts, with values of 0.688 and 0.684 respectively (**Figures 6G, H**), while the AUC values for the XGBoost model are 0.637 and 0.623 (**Figures 6E, F**). Our results demonstrated that the GPI-anchored biosynthesis related genes are valuable as a tool to predict whether a patient has developed severe T cell exhaustion.

### 3.7 Establishment of a prognosis signature related to GPI-anchored biosynthesis

**Figure 7:**
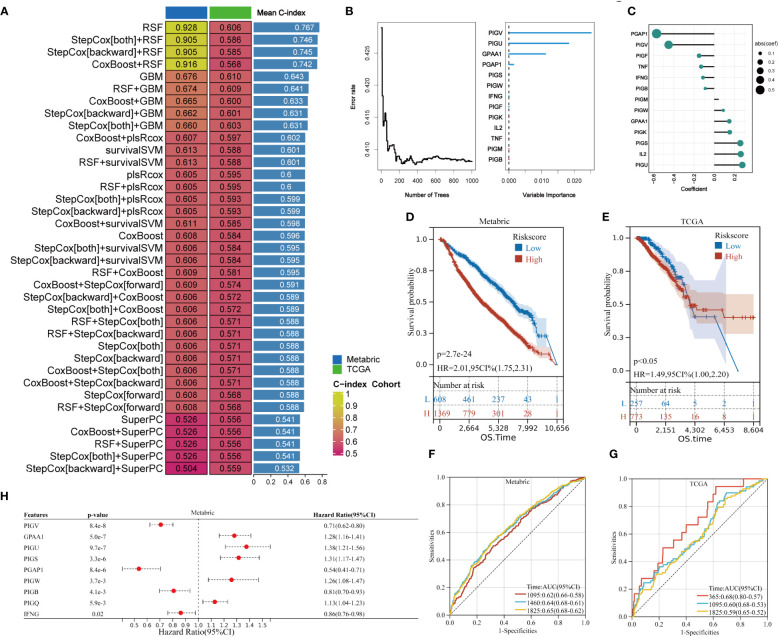
Development and validation of GPIS with 101 machine learning methods. **(A)** The C-index of GPIS for each algorithm combination in Metabric and TCGA datset. **(B)** RandomForestSRC analysis of the genes obtained in RSF. **(C)** Coefficients of 13 genes obtained in RandomForestSRC. **(D, E)** K–M plots for riskscore in Metabric and TCGA sets. **(F, G)** Time-dependent ROC analysis of GPIS, **(F)** AUC for 3-, 4-, and 5-year OS in Metabric set. **(G)** AUC for 1 -, 3 -, and 5-year OS in TCGA set. **(H)** The multivariable Cox regression analysis of GPIS.

To validate the prognostic value of GPI-anchored biosynthesis related genes in BC, we develop a gene signature (GPIS) based on 21 GPI-anchored biosynthesis related genes and the characteristic factors of T cell exhaustion (TNF, IL-2, IFN-γ).The Metabric dataset, with its high number of patients, served as the training set, while the TCGA cohort, with its low number of samples, served as the validation set. We employed a ten-fold cross-validation framework to train and validate 101 predictive models, subsequently determining the C-index for each set. Models with C-index less than 0.5 indicate obvious overfitting problems in the construction process and should be excluded. Interestingly, the optimal model was Random Forest (RSF) with the highest average C-index (0.767) **Figure 7A**. Then, the RandomForestSRC algorithm was utilized to optimize the results of the RSF. **Figure 7B** represents the weight ratio of the characterized genes in GPIS, which shows that PIGV, PIGU, GPAA1, and PGAP1 are the core genes of the model. By weighting the 13 gene expression values and their regression coefficients in RandomForestSRC, a risk score was obtained for each patient (**Figure 7C**; **Supplementary Table 6**). With the optimal thresholds for risk scores determined by the “surviminer” package, patients were classified into high-risk and low-risk groups. K-M curves indicate that patients categorized as high-risk exhibited notably reduced OS duration compared to individuals in the low-risk cohort (**Figures 7D, E**). ROC analysis measured the discrimination of GPIS, and the 3-year, 4-year and 5-year AUC of training dataset were 0.62, 0.64 and 0.65 (**Figure 7F**). The 1-year, 3-year and 5-year AUC in validation dataset were 0.68, 0.60 and 0.59 (**Figure 7G**).The Accuracy of Random Forest model is 0.685, KS score is 0.510, F1 score is 0.645, and Precision is 0.672 (**Supplementary Figure 5**).

### 3.8 GPAA1 and PIGU are associated with poor prognosis and CD8 T cell infiltration in BC

**Figure 8:**
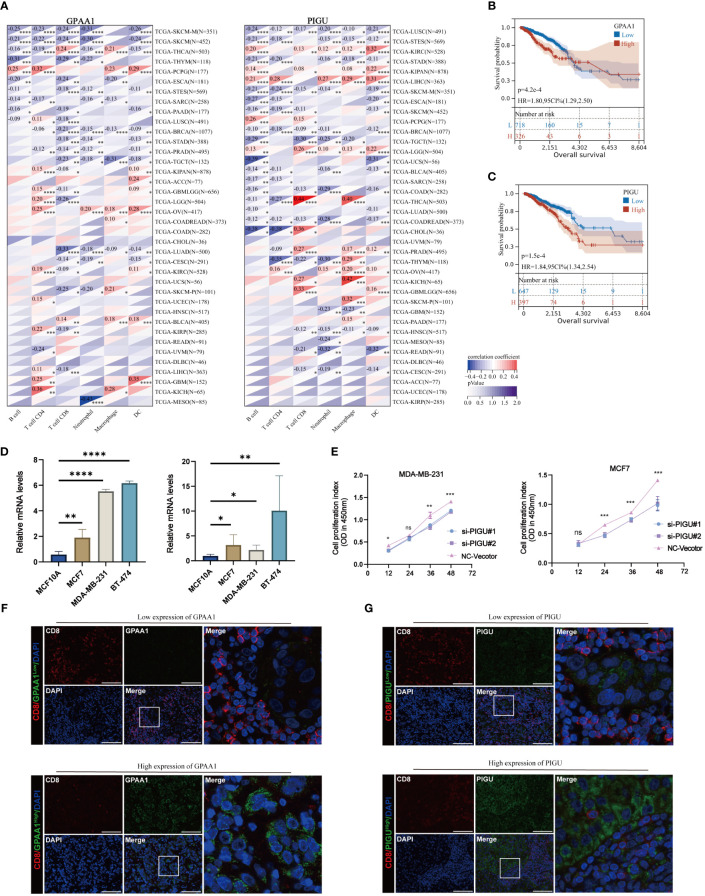
Immunocorrelation analysis and biofunctional validation of GPAA1 and PIGU in BC. **(A)** The correlation of GPAA1 and PIGU with immune cells. **(B, C)** KM plots for OS of GPAA1 and PIGU in TCGA BC cohort. **(D)** The expression of GPAA1 and PIGU in MCF10A, MCF7, MDA-MB-231 and BT-474 cells. **(E)** CCK8 assays in MDA-MB-231 and MCF-7 cells with PIGU-silenced. (ns, p>0.05; *p<0.01; **p< 0.01; ***p< 0.001). **(F, G)** Immunofluorescence (IF) staining of GPAA1, PIGU and CD8.

The multivariable Cox regression analysis revealed that GPAA1, PIGU, PIGS, PIGQ, and PIGW independently serve as risk factors for OS (**Figure 7H**). In the feature weight analysis of the T cell exhaustion diagnostic model and prognosis model, further comprehensive investigation is necessary for GPAA1 and PIGU. We employed the Timer method from the IOBR software package to conduct immune cell infiltration scoring on 9,406 tumor samples covering 38 cancer types within the TCGA pan-cancer dataset. The heat map reveals a strong correlation between GPAA1 and immune infiltration in 32 cancer types, while PIGU exhibits a significant association with immune infiltration in 33 cancer types (**Figure 8A**). The infiltration of CD8+T cells in breast cancer exhibits a negative correlation with the expression levels of GPAA1 and PIGU. In addition, the K-M curve indicates that patients with high expression of GPAA1 and PIGU have a worse OS (**Figures 8B, C**). Compared to normal breast cells (MCF10A), the expression of GPAA1 and PIGU is relatively high in MCF7, MDA-MB-231 and BT-474 cells (**Figure 8D**). SiRNA was employed to downregulate the expression of PIGU in MCF7 and MDA-MB-231 cells, and the ability of PIGU to effectively retard proliferation of MCF7 and MDA-MB-231 cells was confirmed by CCK8 assay (**Figure 8E**).We also employed a multiplex immunofluorescence staining technique to detect the expression levels of GPAA1 and PIGU molecules, as well as the degree of CD8 T cell infiltration in 25 local breast cancer tissues. Surprisingly, In multiple microscopic views, the expression of GPAA1 and PIGU in breast cancer cells was negatively correlated with the expression of CD8 molecules in neighboring lymphocytes (**Figures 8F, G**). When tumor cells high expressed GPAA1 and PIGU, the fluorescence intensity of CD8 molecules in peripheral infiltrating lymphocytes noticeably decreased, indicating a reduction in the number of CD8+ T cells.

## 4 Discussion

Our study demonstrates the dysregulation of GPI-anchored biosynthesis to varying extents in the majority of prevalent tumors. Augmented GPI-anchored biosynthes is not only exhibits a correlation with an unfavorable prognosis in breast cancer but also contributes to the exhaustion of CD8 T cells within the TME. Considering the pivotal role of GPI-anchored biosynthesis in cellular processes (34), we quantified 21 genes associated with GPI-anchored biosynthesis to generate a composite score for assessing the extent of GPI-anchored biosynthesis in tumor patients. Our findings suggest that elevated GPI-anchored biosynthesis functions as a prognostic risk factor in BC patients. Further scoring of the immune status and immune cells suggests that the BC patients with high GPI-anchored biosynthesis had lower levels of immunity, as well as the significant down-regulation of IL2, TNF, and IFN-γ. Studies have shown that during the persistent infection, CD8 T cells undergo a progressive depletion of their effector function and proliferative capacity (35, 36), which is characterized by the upregulation of inhibitory receptors such as PD-1, CTLA4, and TIM-3, as well as impaired production of TNF, IFN-γ, chemokines, and degranulation ability (37–39). Ultimately, the effectiveness of CD8 T cells in inhibiting tumor growth becomes diminished, leading to the immune evasion of tumor cells (40). Interestingly, the characterization of CD8 T cell exhaustion is highly manifested in patients with high GPI-anchored biosynthesis, suggesting that there may be a more severe state of T cell depletion in this patient cluster. This observation motivated us to deep investigate the potential association between CD8 T cell exhaustion levels and GPI-anchored biosynthesis intensity in BC patients.

Referring to the developmental pathway of CD8 T cell exhaustion defined by Beltra et al (41), we identified four distinct patterns of T-cell exhaustion based on the abundance levels of IL-2, TNF, IFN-γ, and CTL expression in the BC patients. As anticipated, the intensity of GPI-anchored biosynthesis in patient clusters increased with the exhaustion of T cells, showing a high correlation between the two. To our surprise, single-cell analysis showed that GPI-anchored biosynthesis was only differentiated in CD8 T cells and over-activated with the exhaustion of CD8 T cells, while this phenomenon was not observed in malignant epithelial and normal breast epithelial cells in TME. This suggests that the enrichment of GPI-anchored biosynthetic activity is an important biological change in the progression of CD8 T cells toward exhaustion.

However, existing studies have not revealed whether abnormalities in the GPI anchoring process in tumors specifically affect the exhaustion of CD8 T cells. Katagiri et al. reported that patients with bone marrow failure (BMF) exhibited T cells deficient in GPI-anchored protein (GPI-AP), and this subset of defective T cells demonstrated heightened susceptibility to immunotherapy (42). More and more studies have proposed that co-expression of some GPI-APs on CD8 T cell can help identify the T cell subsets with weakened function and exhaustion during chronic infection. Such as the co-expression of 2B4, CD160, and KLRG1 with PD-1 on HCV-specific CD8 T cells is associated with intermediate differentiation and impaired function (43, 44). The increased expression of some GPI-anchored inhibitory receptors on T cell surface associated with progressive CD8 T cell exhaustion in BC may be attributed to the boost of GPI anchor biosynthesis.

Our findings suggest a clear mechanistic link between the GPI-anchored biosynthetic pathway and T cell exhaustion, although it has not yet been fully explored. However, based on the existing research results and theoretical hypotheses, we can make some speculations and discuss how GPI-anchored biosynthesis interferes with CD8 T cell exhaustion.1.The role of GPI-anchored proteins in T cell signaling (45): GPI-anchored proteins play a crucial role in signal transduction and cell-cell interactions on the cell membrane. There may exist GPI-anchored proteins that play key roles in T cell activation and maintenance of function. Therefore, abnormalities in GPI-anchored protein biosynthesis may affect the expression or function of these proteins, leading to T cell dysfunction. 2.Interaction of GPI-anchored proteins with immune regulatory factors (46): GPI-anchored proteins may interact with immune regulatory factors, influencing the immune regulatory function of T cells. Abnormal GPI-anchored protein biosynthesis may disrupt these interactions, thereby affecting T cell activation, proliferation, and effector function.3. The role of GPI-anchored proteins in T cell metabolism regulation (47): GPI-anchored proteins may participate in regulating the metabolic processes of T cells, including energy metabolism and signaling pathways. Abnormal GPI-anchored protein biosynthesis may impact the normal functioning of these metabolic pathways, resulting in T cell dysfunction and exhaustion. 4.Association of GPI-anchored proteins with immune checkpoints (47): GPI-anchored proteins may be associated with immune checkpoint molecules, influencing the balance between T cell activation and suppression. Abnormal GPI-anchored protein biosynthesis may alter the expression or function of these immune checkpoints, leading to T cell dysfunction and immune exhaustion.

Furthermore, the persistent presence of a metabolic state, such as an inflammatory state, in tumor patients can directly influence the tumor microenvironment. Research indicates that immune cells in the tumor microenvironment can exhibit metabolic plasticity based on changes in the TME (48). For example, MRC1+ CCL18+ macrophages in colorectal cancer show enrichment in oxidative phosphorylation, while macrophages in liver metastasis primarily rely on amino acid metabolism (49). This metabolic adaptability directly impacts the function, activation state, and response of immune cells to the tumor (50). Metabolites derived from glutamine metabolism, for instance, can promote T cell-driven immunosuppressive programs (51).In breast cancer patients, the increased expression of some GPI-anchored inhibitory receptors on the surface of CD8+ T cells during T cell exhaustion may be related to cellular responses to changes in the TME, leading to metabolic reprogramming and potentially promoting increased GPI-anchored protein biosynthesis.

Overall, the potential mechanistic links between GPI-anchored protein biosynthesis and T cell dysfunction may involve multiple aspects, including signal transduction, immune regulation, metabolic regulation, and immune checkpoints. Future research can further explore these mechanistic connections to uncover the specific role of GPI-anchored protein biosynthesis in T cell function regulation, providing a deeper theoretical basis for the development of novel immunotherapy strategies.

In addition to suggesting enhanced GPI-anchored biosynthesis, which is associated with CD8 T cell exhaustion and poor prognosis in BC, we also observed different perturbations of expression of 21 genes involved in GPI-anchored biosynthetic processes during the transformation process of CD8 T cell exhaustion. With the further establishment of BC prediction models for GPI-anchored biosynthesis-related genes, the role of GPAA1 and PIGU are pivotal in T cell exhaustion diagnosis and patient prognosis, suggesting the potential utility of GPI-anchored biosynthesis related genes as biomarkers for diagnosing T cell depletion status and predicting prognosis in BC patients.

In fact, it has been shown that GPAA1 and PIGU are defined as oncogenes in a variety of cancers. For example, GPAA1 facilitates the advancement of gastric cancer by increasing the expression of GPI-anchored proteins and augmenting the ERBB signaling pathway (52). High expression of GPAA1 exacerbates the progression of hepatocellular carcinoma (53). And PIGU was recognized as an oncogenic factor of bladder cancer in 2004 (54). This opens a new door that GPI-anchored bioanabolic activity itself may be tumorigenic. We also found that GPAA1 and PIGU are highly amplified in breast cancer cell lines (55), and inhibiting the expression of PIGU has a negative impact on cell proliferation. Not only that, BC tissues with high expression of GPAA1 and PIGU exhibited suppression of peripheral CD8 T cell infiltration.

In conclusion, our study provides a valuable insight into T cell exhaustion in BC patients. We strongly recommend the inclusion of GPI-anchor biosynthesis metabolic pathway as a potential risk factor for tumorigenesis and progression of T-cell exhaustion. And we advocate for increased attention to be directed toward the aspect of GPI-anchor biosynthesis in future research endeavors. In-depth exploration of the impact of GPI-anchor biosynthesis on T cell function may contributes to uncovering mechanisms of tumor immune tolerance and provides a theoretical basis for the development of novel immunotherapy strategies.

## Data availability statement

The datasets presented in this study can be found in online repositories. The names of the repository/repositories and accession number(s) can be found in the article/**Supplementary Material**.

## Ethics statement

The studies involving humans were approved by Clinical Research Ethics Committee of the First Affiliated Hospital of Wenzhou Medical University. The studies were conducted in accordance with the local legislation and institutional requirements. The participants provided their written informed consent to participate in this study. Ethical approval was not required for the studies on animals in accordance with the local legislation and institutional requirements because only commercially available established cell lines were used.

## Author contributions

HW: Writing – original draft, Writing – review & editing, Conceptualization, Data curation, Formal analysis, Investigation, Methodology, Project administration, Resources, Software, Supervision, Validation. ZXW: Writing – original draft, Writing – review & editing, Conceptualization, Investigation, Software. DY: Writing – original draft, Writing – review & editing. HL: Conceptualization, Investigation, Software, Writing – original draft, Writing – review & editing. ZQW: Conceptualization, Data curation, Investigation, Writing – review & editing. JB: Validation, Conceptualization, Investigation, Project administration, Writing – review & editing. YC: Data curation, Formal analysis, Project administration, Writing – original draft, Writing – review & editing. BC: Supervision, Conceptualization, Investigation, Software, Writing – review & editing. SX: Supervision, Writing – review & editing, Methodology, Software, Data curation, Project administration. EX: Software, Supervision, Validation, Visualization, Writing – review & editing. XD: Conceptualization, Data curation, Formal analysis, Funding acquisition, Investigation, Methodology, Project administration, Resources, Software, Supervision, Validation, Visualization, Writing – original draft, Writing – review & editing.

## References

[1] Britt KL Cuzick J Phillips KA. Key steps for effective breast cancer prevention. Nat Rev Cancer. (2020) 20:417–36. doi: 10.1038/s41568-020-0266-x 32528185

[2] Sung H Ferlay J Siegel R. L Laversanne M Soerjomataram I Jemal A. Global cancer statistics 2020: GLOBOCAN estimates of incidence and mortality worldwide for 36 cancers in 185 countries. CA Cancer J Clin. (2021) 71:209–49. doi: 10.3322/caac.21660 33538338

[3] Kennedy LB Salama AKS. A review of cancer immunotherapy toxicity. CA Cancer J Clin. (2020) 70:86–104. doi: 10.3322/caac.21596 31944278

[4] Stanton SE Adams S Disis ML. Variation in the incidence and magnitude of tumor-infiltrating lymphocytes in breast cancer subtypes: A systematic review. JAMA Oncol. (2016) 2:1354–60. doi: 10.1001/jamaoncol.2016.1061 27355489

[5] McLane LM Abdel-Hakeem MS Wherry EJ. CD8 T cell exhaustion during chronic viral infection and cancer. Annu Rev Immunol. (2019) 37:457–95. doi: 10.1146/annurev-immunol-041015-055318 30676822

[6] Kurachi M. CD8(+) T cell exhaustion. Semin Immunopathol. (2019) 41:327–37. doi: 10.1007/s00281-019-00744-5 30989321

[7] Wang Q Qin Y Li B. CD8(+) T cell exhaustion and cancer immunotherapy. Cancer Lett. (2023) 559:216043. doi: 10.1016/j.canlet.2022.216043 36584935

[8] Takeda J Kinoshita T. GPI-anchor biosynthesis. Trends Biochem Sci. (1995) 20:367–71. doi: 10.1016/S0968-0004(00)89078-7 7482705

[9] Yeh ET Kamitani T Chang HM. Biosynthesis and processing of the glycosylphosphatidylinositol anchor in mammalian cells. Semin Immunol. (1994) 6:73–80. doi: 10.1006/smim.1994.1011 8054538

[10] Brown D Waneck GL. Glycosyl-phosphatidylinositol-anchored membrane proteins. J Am Soc Nephrol. (1992) 3:895–906. doi: 10.1681/ASN.V34895 1450366

[11] Hill A DeZern AE Kinoshita T Brodsky RA. Paroxysmal nocturnal haemoglobinuria. Nat Rev Dis Primers. (2017) 3:17028. doi: 10.1038/nrdp.2017.28 28516949 PMC7879566

[12] Krawitz PM Schweiger MR Rödelsperger C Marcelis C Kölsch U Meisel C. Identity-by-descent filtering of exome sequence data identifies PIGV mutations in hyperphosphatasia mental retardation syndrome. Nat Genet. (2010) 42:827–9. doi: 10.1038/ng.653 20802478

[13] Guo Z Kundu S. Recent research progress in glycosylphosphatidylinositol-anchored protein biosynthesis, chemical/chemoenzymatic synthesis, and interaction with the cell membrane. Curr Opin Chem Biol. (2024) 78:102421. doi: 10.1016/j.cbpa.2023.102421 38181647 PMC10922524

[14] Zhang JY Wang XM Xing X Xu Z Zhang C Song JW. Single-cell landscape of immunological responses in patients with COVID-19. Nat Immunol. (2020) 21:1107–18. doi: 10.1038/s41590-020-0762-x 32788748

[15] Loertscher R Lavery P. The role of glycosyl phosphatidyl inositol (GPI)-anchored cell surface proteins in T-cell activation. Transpl Immunol. (2002) 9:93–6. doi: 10.1016/S0966-3274(02)00013-8 12180852

[16] Dolina JS Van Braeckel-Budimir N Thomas GD Salek-Ardakani S. CD8(+) T cell exhaustion in cancer. Front Immunol. (2021) 12:715234. doi: 10.3389/fimmu.2021.715234 34354714 PMC8330547

[17] Mii S Enomoto A Shiraki Y Taki T Murakumo Y Takahashi M. CD109: a multifunctional GPI-anchored protein with key roles in tumor progression and physiological homeostasis. Pathol Int. (2019) 69:249–59. doi: 10.1111/pin.12798 31219232

[18] Yip C Foidart P Noël A Sounni NE. MT4-MMP: the GPI-anchored membrane-type matrix metalloprotease with multiple functions in diseases. Int J Mol Sci. (2019) 20:354. doi: 10.3390/ijms20020354 30654475 PMC6359745

[19] Gamage DG Hendrickson TL. GPI transamidase and GPI anchored proteins: oncogenes and biomarkers for cancer. Crit Rev Biochem Mol Biol. (2013) 48:446–64. doi: 10.3109/10409238.2013.831024 23978072

[20] Xia J Wishart DS. MSEA: a web-based tool to identify biologically meaningful patterns in quantitative metabolomic data. Nucleic Acids Res. (2010) 38:W71–7. doi: 10.1093/nar/gkq329 PMC2896187 20457745

[21] Takahashi H Kawaguchi T Yan L Peng X Qi Q Morris LGT. Immune cytolytic activity for comprehensive understanding of immune landscape in hepatocellular carcinoma. Cancers (Basel). (2020) 12:1221. doi: 10.3390/cancers12051221 32414098 PMC7281225

[22] Wu Z Wang Z Wu H Zheng N Huang D Huang Z. The pan-cancer multi-omics landscape of key genes of sialylation combined with RNA-sequencing validation. Comput Biol Med. (2023) 166:107556. doi: 10.1016/j.compbiomed.2023.107556 37801920

[23] Rich JT Neely JG Paniello RC Voelker CC Nussenbaum B Wang EW. A practical guide to understanding Kaplan-Meier curves. Otolaryngol Head Neck Surg. (2010) 143:331–6. doi: 10.1016/j.otohns.2010.05.007 PMC3932959 20723767

[24] Shen W Song Z Zhong X Huang M Shen D Gao P. Sangerbox: A comprehensive, interaction-friendly clinical bioinformatics analysis platform. iMeta. (2022) 1:e36. doi: 10.1002/imt2.36 38868713 PMC10989974

[25] Newman AM Liu CL Green MR Gentles AJ Feng W Xu Y. Robust enumeration of cell subsets from tissue expression profiles. Nat Methods. (2015) 12:453–7. doi: 10.1038/nmeth.3337 PMC4739640 25822800

[26] Yoshihara K Shahmoradgoli M Martínez E Vegesna R Kim H Torres-Garcia W. Inferring tumour purity and stromal and immune cell admixture from expression data. Nat Commun. (2013) 4:2612. doi: 10.1038/ncomms3612 24113773 PMC3826632

[27] Wherry EJ. T cell exhaustion. Nat Immunol. (2011) 12:492–9. doi: 10.1038/ni.2035 21739672

[28] Wherry EJ Kurachi M. Molecular and cellular insights into T cell exhaustion. Nat Rev Immunol. (2015) 15:486–99. doi: 10.1038/nri3862 PMC4889009 26205583

[29] Seiler M Huang CC Szalma S Bhanot G. ConsensusCluster: a software tool for unsupervised cluster discovery in numerical data. Omics. (2010) 14:109–13. doi: 10.1089/omi.2009.0083 20141333

[30] Chen K Wang Y Hou Y Wang Q Long D Liu X. Single cell RNA-seq reveals the CCL5/SDC1 receptor-ligand interaction between T cells and tumor cells in pancreatic cancer. Cancer Lett. (2022) 545:215834. doi: 10.1016/j.canlet.2022.215834 35917973

[31] Trapnell C Cacchiarelli D Grimsby J Pokharel P Li S Morse M. The dynamics and regulators of cell fate decisions are revealed by pseudotemporal ordering of single cells. Nat Biotechnol. (2014) 32:381–6. doi: 10.1038/nbt.2859 PMC4122333 24658644

[32] Liu Z Liu L Weng S Guo C Dang Q Xu H. Machine learning-based integration develops an immune-derived lncRNA signature for improving outcomes in colorectal cancer. Nat Commun. (2022) 13:816. doi: 10.1038/s41467-022-28421-6 35145098 PMC8831564

[33] Gao J Liang Y Wang L. Shaping polarization of tumor-associated macrophages in cancer immunotherapy. Front Immunol. (2022) 13:888713. doi: 10.3389/fimmu.2022.888713 35844605 PMC9280632

[34] Kinoshita T Fujita M Maeda Y. Biosynthesis, remodelling and functions of mammalian GPI-anchored proteins: recent progress. J Biochem. (2008) 144:287–94. doi: 10.1093/jb/mvn090 18635593

[35] Preglej T Ellmeier W. CD4(+) cytotoxic T cells - phenotype, function and transcriptional networks controlling their differentiation pathways. Immunol Lett. (2022) 247:27–42. doi: 10.1016/j.imlet.2022.05.001 35568324

[36] Wik JA Skålhegg BS. T cell metabolism in infection. Front Immunol. (2022) 13:840610. doi: 10.3389/fimmu.2022.840610 35359994 PMC8964062

[37] Anderson AC Joller N Kuchroo VK. Lag-3, tim-3, and TIGIT: co-inhibitory receptors with specialized functions in immune regulation. Immunity. (2016) 44:989–1004. doi: 10.1016/j.immuni.2016.05.001 27192565 PMC4942846

[38] Huang YH Zhu C Kondo Y Anderson AC Gandhi A Russell A. CEACAM1 regulates TIM-3-mediated tolerance and exhaustion. Nature. (2015) 517:386–90. doi: 10.1038/nature13848 PMC4297519 25363763

[39] Ju F Luo Y Lin C Jia X Xu Z Tian R. Oncolytic virus expressing PD-1 inhibitors activates a collaborative intratumoral immune response to control tumor and synergizes with CTLA-4 or TIM-3 blockade. J Immunother Cancer. (2022) 10:e004762. doi: 10.1136/jitc-2022-004762 35688558 PMC9189843

[40] Farhood B Najafi M Mortezaee K. CD8(+) cytotoxic T lymphocytes in cancer immunotherapy: A review. J Cell Physiol. (2019) 234:8509–21. doi: 10.1002/jcp.27782 30520029

[41] Beltra JC Manne S Abdel-Hakeem MS Kurachi M Giles JR Chen Z. Developmental relationships of four exhausted CD8(+) T cell subsets reveals underlying transcriptional and epigenetic landscape control mechanisms. Immunity. (2020) 52:825–841.e8. doi: 10.1016/j.immuni.2020.04.014 32396847 PMC8360766

[42] Katagiri T Qi Z Ohtake S Nakao S. GPI-anchored protein-deficient T cells in patients with aplastic anemia and low-risk myelodysplastic syndrome: implications for the immunopathophysiology of bone marrow failure. Eur J Haematol. (2011) 86:226–36. doi: 10.1111/j.1600-0609.2010.01563.x 21166881

[43] Bengsch B Ohtani T Khan O Setty M Manne S O'Brien S. Epigenomic-guided mass cytometry profiling reveals disease-specific features of exhausted CD8 T cells. Immunity. (2018) 48:1029–1045.e5. doi: 10.1016/j.immuni.2018.04.026 29768164 PMC6010198

[44] Jhunjhunwala S Hammer C Delamarre L. Antigen presentation in cancer: insights into tumour immunogenicity and immune evasion. Nat Rev Cancer. (2021) 21:298–312. doi: 10.1038/s41568-021-00339-z 33750922

[45] Malek TR Fleming TJ Codias EK. Regulation of T lymphocyte function by glycosylphosphatidylinositol (GPI)-anchored proteins. Semin Immunol. (1994) 6:105–13. doi: 10.1006/smim.1994.1015 8054537

[46] Lakhan SE Sabharanjak S De A. Endocytosis of glycosylphosphatidylinositol-anchored proteins. J BioMed Sci. (2009) 16:93. doi: 10.1186/1423-0127-16-93 19832981 PMC2764642

[47] Wang X Liu M Zhang J Brown NK Zhang P Zhang Y. CD24-Siglec axis is an innate immune checkpoint against metaflammation and metabolic disorder. Cell Metab. (2022) 34:1088–1103.e6. doi: 10.1016/j.cmet.2022.07.005 35921817 PMC9393047

[48] Wu Y Ma J Yang X Nan F Zhang T Ji S. Neutrophil profiling illuminates anti-tumor antigen-presenting potency. Cell. (2024) 187:1422–1439.e24. doi: 10.1016/j.cell.2024.02.005 38447573

[49] Ma J Wu Y Ma L Yang X Zhang T Song G. A blueprint for tumor-infiltrating B cells across human cancers. Science. (2024) 384:eadj4857. doi: 10.1126/science.adj4857 38696569

[50] Wu Y Yang S Ma J Chen Z Song G Rao D. Spatiotemporal immune landscape of colorectal cancer liver metastasis at single-cell level. Cancer Discovery. (2022) 12:134–53. doi: 10.1158/2159-8290.CD-21-0316 34417225

[51] Wu Y Zou Q Jiang P Gao Q. Tumor-host cometabolism collaborates to shape cancer immunity. Cancer Discovery. (2024) 14:653–7. doi: 10.1158/2159-8290.CD-23-1509 38571418

[52] Zhang XX Ni B Li Q Hu LP Jiang SH Li RK. GPAA1 promotes gastric cancer progression via upregulation of GPI-anchored protein and enhancement of ERBB signalling pathway. J Exp Clin Cancer Res. (2019) 38:214. doi: 10.1186/s13046-019-1218-8 31118109 PMC6532258

[53] Ho JC Cheung ST Patil M Chen X Fan ST. Increased expression of glycosyl-phosphatidylinositol anchor attachment protein 1 (GPAA1) is associated with gene amplification in hepatocellular carcinoma. Int J Cancer. (2006) 119:1330–7. doi: 10.1002/ijc.22005 16642471

[54] Guo Z Linn JF Wu G Anzick SL Eisenberger CF Halachmi S. CDC91L1 (PIG-U) is a newly discovered oncogene in human bladder cancer. Nat Med. (2004) 10:374–81. doi: 10.1038/nm1010 15034568

[55] Wu G Guo Z Chatterjee A Huang X Rubin E Wu F. Overexpression of glycosylphosphatidylinositol (GPI) transamidase subunits phosphatidylinositol glycan class T and/or GPI anchor attachment 1 induces tumorigenesis and contributes to invasion in human breast cancer. Cancer Res. (2006) 66:9829–36. doi: 10.1158/0008-5472.CAN-06-0506 17047043

