# Supplementary figures and images for: Glycosylphosphatidylinositol anchor biosynthesis pathway-based biomarker identification with machine learning for prognosis and T cell exhaustion status prediction in breast cancer
Source: Front Immunol. 2024 Jul 2;15:1392940. doi: 10.3389/fimmu.2024.1392940 (PMC11249538; doi:10.3389/fimmu.2024.1392940)

consensus matrix k=4

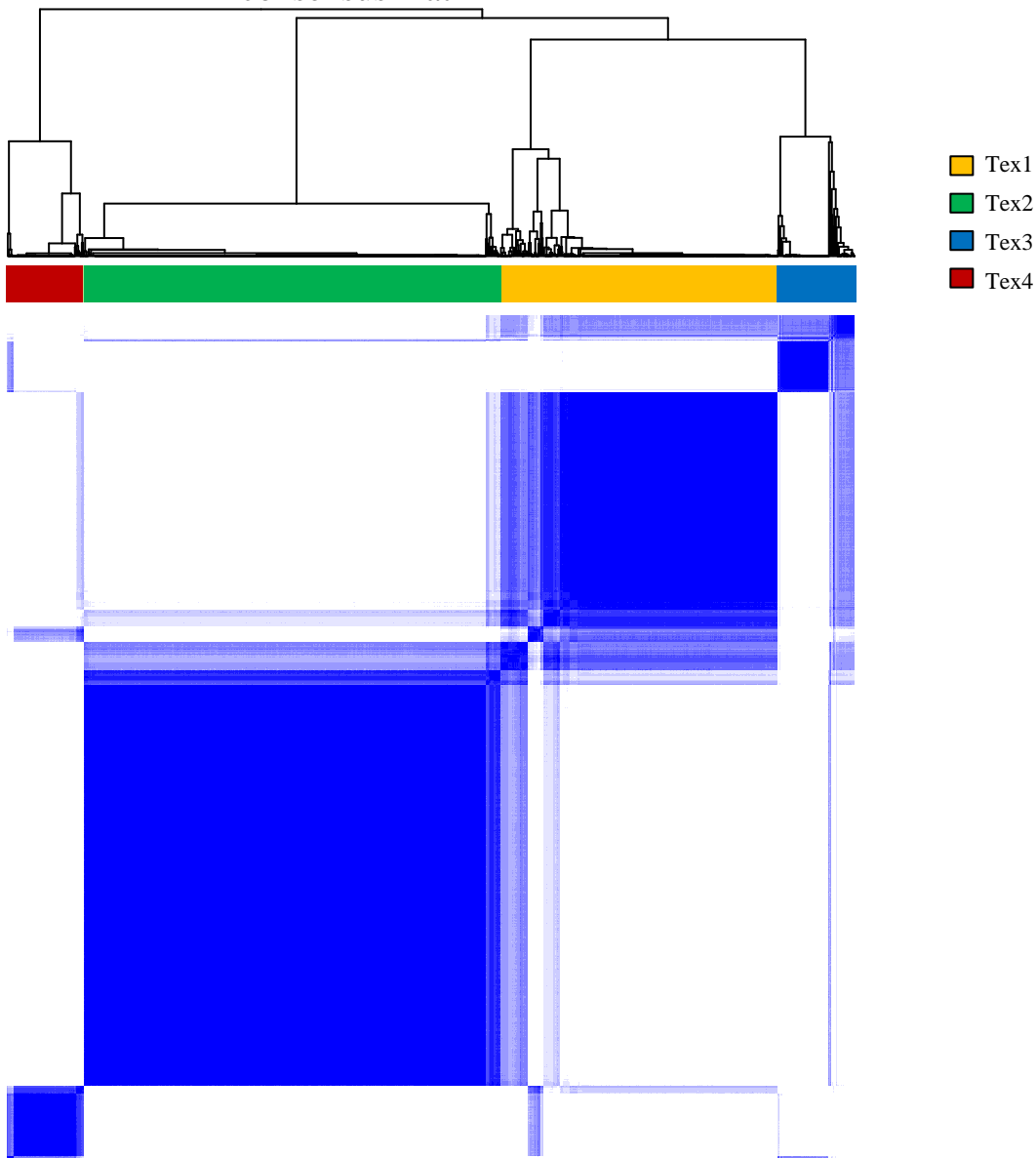

Supplement: Supplementary file 1 [file DataSheet_1.zip › FigureS1.pdf]

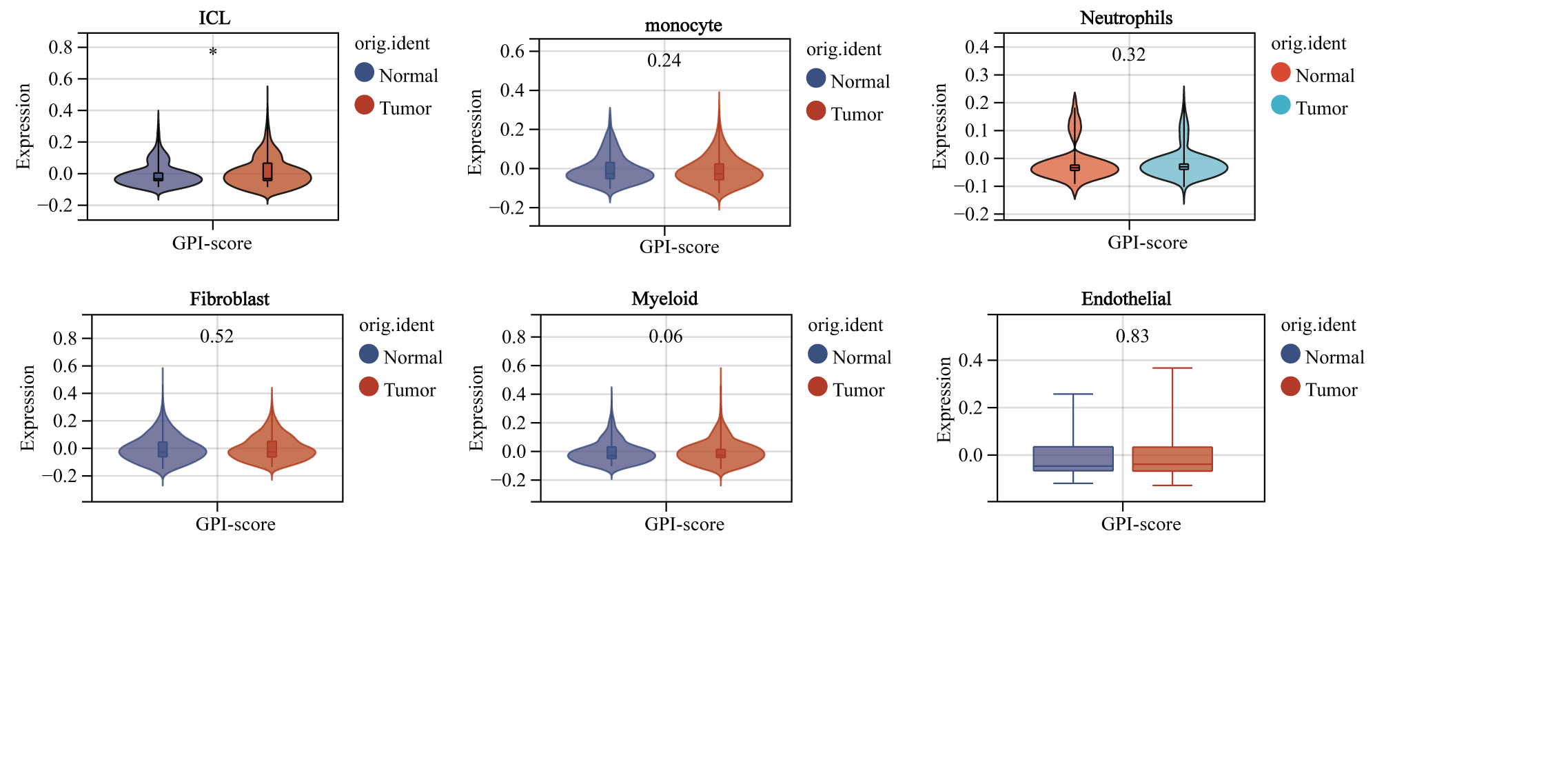

Supplement: Supplementary file 1 [file DataSheet_1.zip › FigureS2.tif]

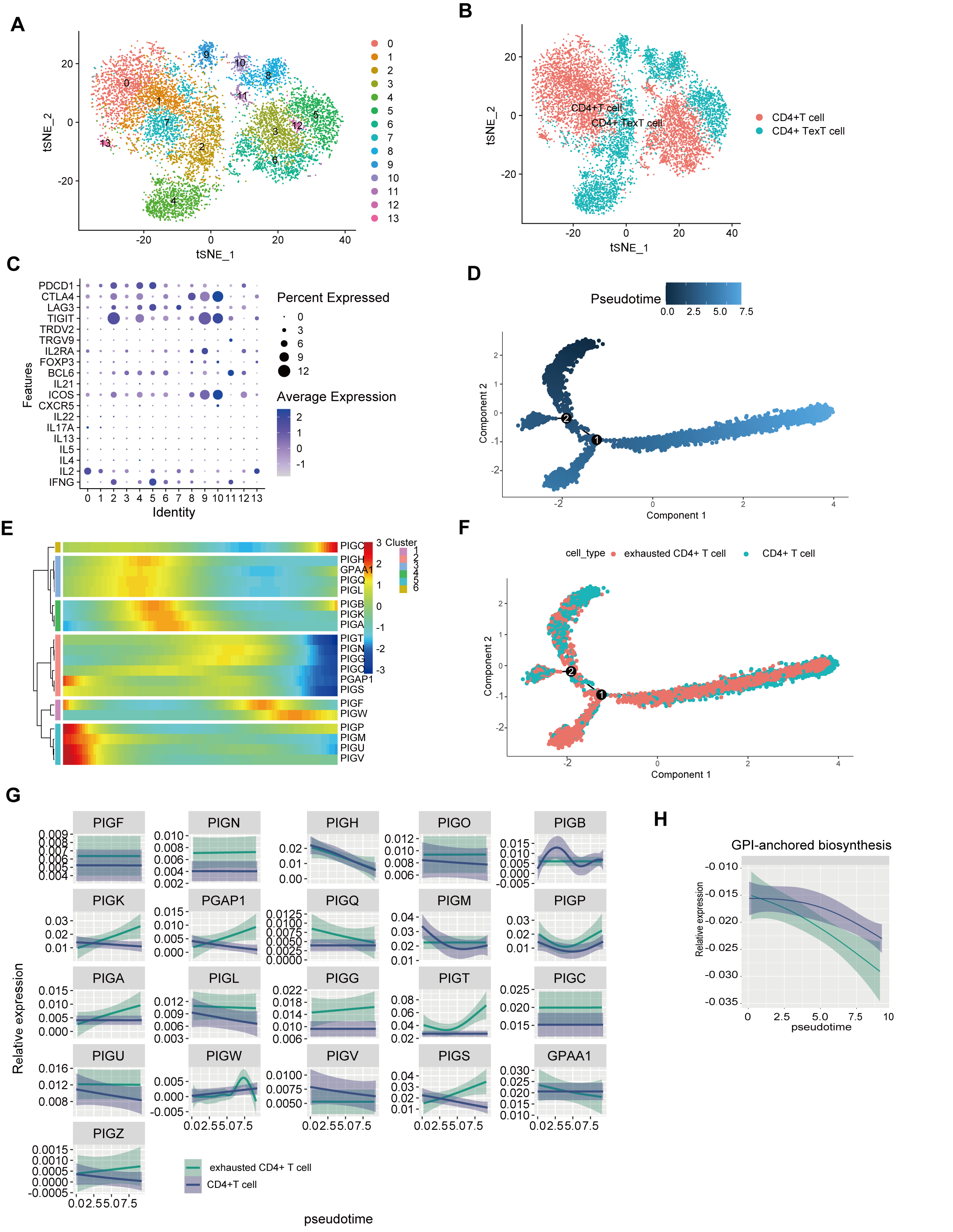

Supplement: Supplementary file 1 [file DataSheet_1.zip › FigureS3.tif]

## GPIS

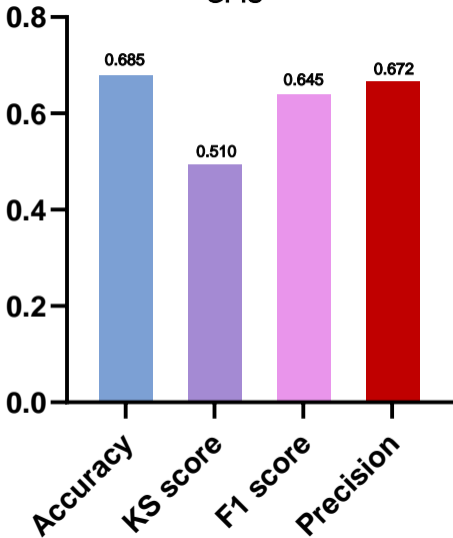

Supplement: Supplementary file 1 [file DataSheet_1.zip › FigureS5.pdf]
